# Small Bowel Recurrent Intussusception Status Post Gastric Bypass

**DOI:** 10.7759/cureus.71230

**Published:** 2024-10-10

**Authors:** Yasmine Ghattas, Brittany L Perry, Danis Lester, Aidan Charles, Darwin Ang

**Affiliations:** 1 Medicine, University of Central Florida College of Medicine, Orlando, USA; 2 General Surgery, Hospital Corporation of America (HCA) Healthcare, Ocala, USA; 3 Trauma, Hospital Corporation of America (HCA) Healthcare, Ocala, USA; 4 Trauma, University of South Florida, Tampa, USA

**Keywords:** antiperistaltic anastomosis, gastric bypass, intussusception, intussusception reduction, isoperistaltic small bowel anastomosis, jejunal intussusception, jejunojejunostomy, recurrent intussusception

## Abstract

Intussusception is an uncommon problem in the adult population. Interestingly, it is also a rare late complication after gastric bypass. We report the case of a 73-year-old woman with a history of Roux-en-Y gastric bypass (RYGB) in 2004 complicated by jejunal intussusception in 2011 for which a small bowel resection was performed. The patient presented to the emergency department (ED) with intractable abdominal pain and nausea. The diagnosis of intussusception was made by abdominal CT scan. Initial laparoscopic reduction of the small bowel intussusception resulted in recurrence within the first 24 hours, and exploratory laparotomy with isoperistaltic small bowel anastomosis was then performed. Careful consideration in isolated reduction of uncomplicated recurrent small bowel intussusception is important. Our case describes an unsuccessful simple reduction in a patient with recurrent intussusception status post gastric bypass. While reduction is more conservative, in patients with recurrent intussusception, it is important to consider a reconstructive option such as resection and reconstruction of the jejunojejunostomy, as it appears to be associated with acceptable complication rates and the lowest risk of recurrence.

## Introduction

Data from 2020 reports that there are approximately 200,000 bariatric procedures performed each year in the US of which about 20% are Roux-en-Y gastric bypass (RYGB) [1]. Intussusception is generally rare in adults and typically secondary to pathologic lead point (i.e., malignancy, polyps, inflammatory processes) [2]. However, intussusception can also rarely present as a late complication of RYGB, with a reported incidence of 4.7% [3].

Clinical diagnosis of intussusception is limited due to nonspecific symptoms, physical exam findings, and lab findings. Intussusception is typically diagnosed via radiogram, contrast-enhanced computerized tomography (CECT), and/or abdominal ultrasounds (US). Target signs on axial views can sometimes be observed on abdominal ultrasounds or CECT which shows the invaginated portion of the bowel appearing as rings on a target. Contrast or pneumatic enema using US or fluoroscopy can be used as a confirmatory test and shows interruption of contrast or air at the site of invagination.

This paper presents the case of a patient with a past surgical history of RYGB complicated by small bowel resection secondary to jejunal intussusception who presented with recurrent jejunal intussusception. Our paper presents the first case of recurrent intussusception spanning decades after initial RYGB. 

## Case presentation

Clinical course

A 73-year-old Caucasian female status post RYGB 19 years prior to admission, with small bowel resection due to small bowel intussusception 13 years prior to admission, presented with acute-onset abdominal pain. Her pain started the previous evening and was nonspecific but primarily localized to the right lower quadrant of the abdomen. The patient reported a four-day history of diarrhea followed by two days of obstipation prior to admission.

She reported a 108-pound weight loss in the first year after her RYGB (change in BMI of 20.41 units, from 42.51 to 22.1). She has maintained her weight in the 53-56 kg range since the initial surgery. The patient had a past medical history of hypertension, Hashimoto’s thyroiditis, restless leg syndrome, and pulmonary embolism (secondary to contraceptive use). Her family history was noncontributory, and she denied any alcohol, tobacco, or recreational drug usage.

Objective and physical exam 

On initial presentation, the patient’s vitals included a temperature of 37.1 °C, blood pressure of 138/90 mmHg, heart rate of 97 bpm, respiratory rate of 18 breaths per minute, and pulse oximetry of 99% on room air. Physical examination revealed well-healed scars from prior abdominal surgical incisions without hernias. There was moderate diffuse abdominal tenderness with guarding but no rebound tenderness. 

Diagnostic studies and clinical decision-making

**Figure 1:**
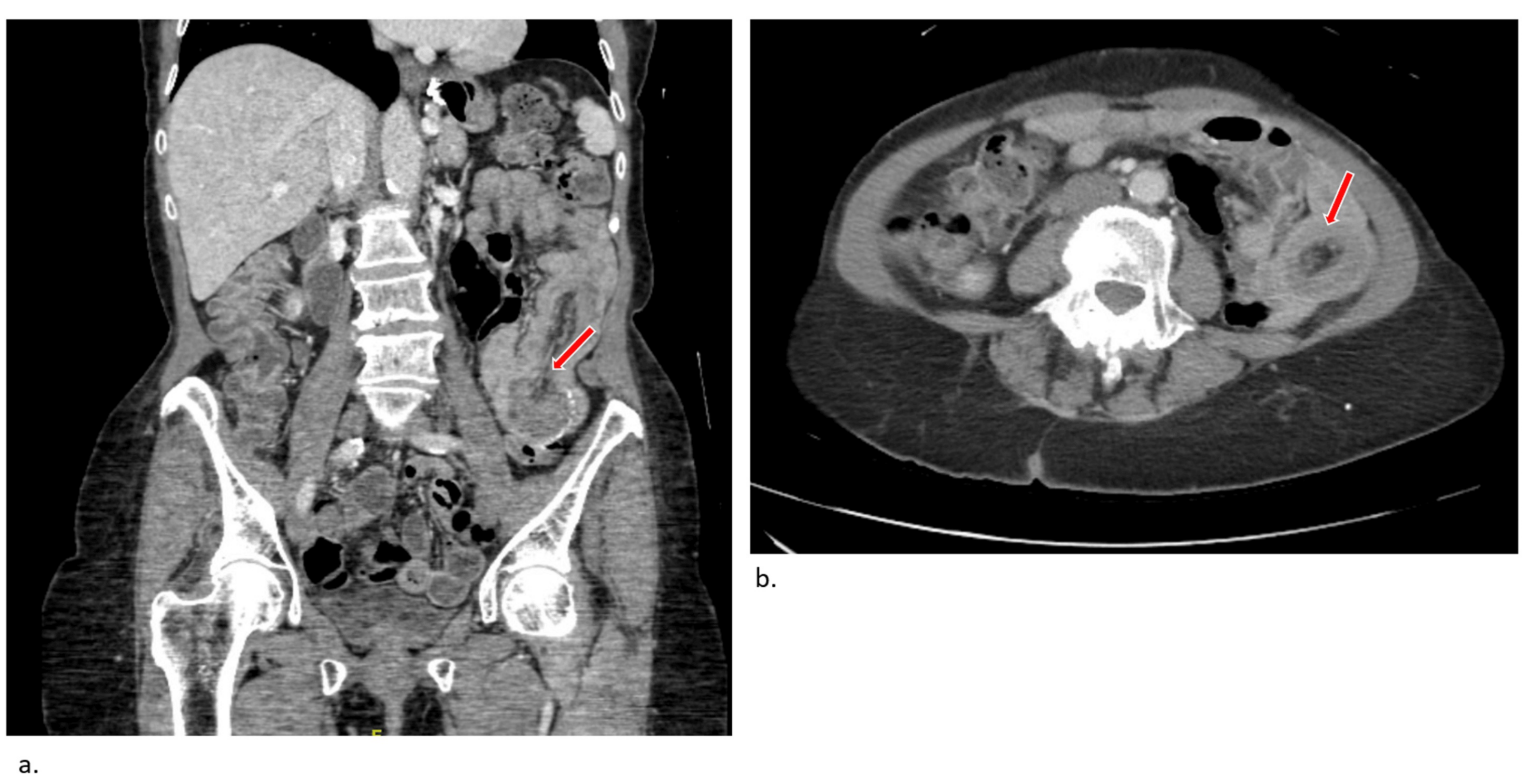
Abdominal CECT showing postsurgical changes from gastric bypass with a retrograde intussusception at the jejunal anastomosis in the left side of the abdomen with no evidence of obstruction. (a) Coronal view with arrow indicating a transition point and (b) axial view with a characteristic "donut" sign showing small bowel intussusception CECT: Contrast-enhanced computerized tomography Images reproduced with patient permission.

The patient underwent abdominal CECT that showed postsurgical changes from gastric bypass with a retrograde intussusception at the jejunal anastomosis in the left side of the abdomen with no evidence of obstruction (Figure 1).

Treatment

The patient underwent diagnostic laparoscopy the day of her admission. Jejunal intussusception was observed at the site of the jejunojejunostomy and noted to measure at least 10 cm. The bowel appeared healthy and without ischemic change. Given prior reports of successful simple reduction, reduction without enteropexy was performed. No complications occurred during the operation.

The patient was admitted for observation. Postoperative day one, she started complaining of worsening epigastric pain that was different from the pain when she had first arrived. This pain was unrelieved by a multimodal pain regiment. She had no overt signs of peritonitis but endorsed subjective fevers. Abdominal CT revealed high-grade mechanical obstruction of the proximal small bowel likely secondary to a large intussusception in the left abdomen, and immediate surgical evaluation was completed. 

The patient was taken back to the operating room postoperative day one for an exploratory laparotomy. Proximal jejunal intussusception of the roux limbs was observed. Isoperistaltic small bowel anastomosis was pursued, and defects were made in the small bowel mesentery proximally and distally to the anastomosis. A gastrojejunal tube was placed, and dense adhesions were lysed around the stomach. During the operation a small splenic capsular tear occurred, which was controlled with electrocautery.

Outcome and follow-up

The patient was progressing well and was able to tolerate oral intake without difficulty. Her hospitalization was complicated by unforeseen pulmonary complications and surgical site infection. Patient ultimately elected to pursue hospice for further care.

## Discussion

Gastric bypass is a relatively common procedure in the United States. Although rare, late complications of RYGB such as jejunal intussusception are imperative to recognize and treat in a timely manner due to the consequences of a missed diagnosis, such as bowel necrosis, sepsis, or death [4]. 

**Figure 2:**
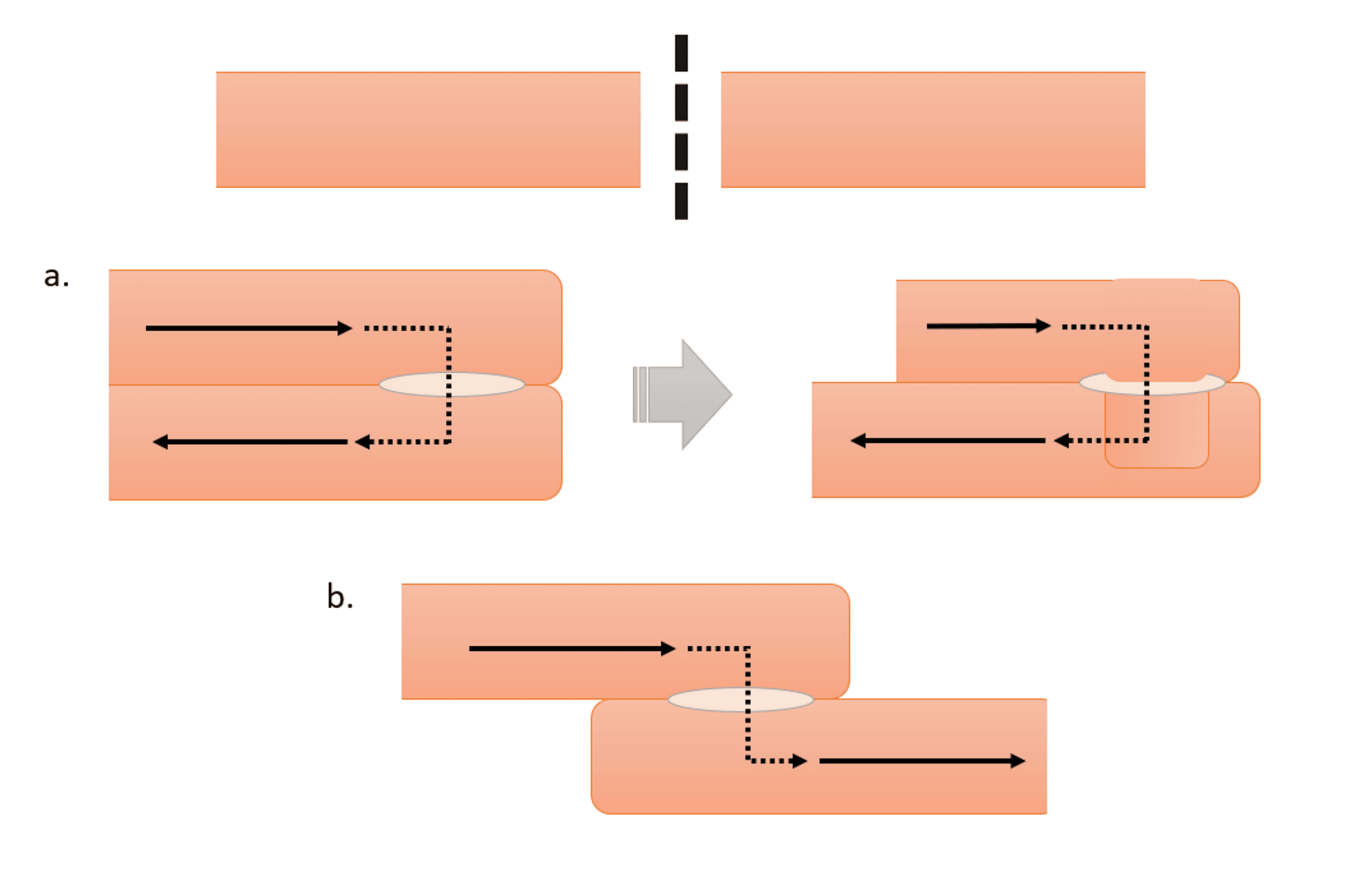
Bowel resection and peristaltic direction after (a) antiperistaltic anastomosis, with potential physiologic encouragement of intussusception or (b) isoperistaltic anastomosis Original image by authors

Although the exact pathophysiology behind jejunal intussusception status post RYGB is not yet elucidated, it is likely multifactorial [5]. The staple/suture line may act as a lead point for intussusception [5,6]. During construction of the Roux limb, the distal jejunum becomes separated from the duodenal pacemaker cells due to jejunal transection. As small bowel motility is initiated in the duodenum, this separation may cause ectopic pacemakers to arise in the Roux limb, producing anterograde and/or retrograde peristalsis [6]. As multiple studies demonstrate significant weight loss at the time of patient presentation, weight loss leading to thinning of the mesentery may provide increased mobility around the jejunojejunal anastomosis [7,8]. It has been suggested that in antiperistaltic anastomoses, the natural antiperistaltic (retrograde) movement leads to the telescoping of the common limb into the jejunal anastomosis (Figure 2) [9]. Others report intussusception occurring at both isoperistaltic and antiperistaltic RYGB sites [8]. 

This is the first case in the literature describing failure of reduction and recurrence of intussusception within 24 hours. Prior studies have suggested that if the bowel is not ischemic and is amenable to resection, as was the case in our patient, conservative surgical management (i.e., reduction with or without enteropexy) is a feasible option, as conservative management is associated with decreased hospital stays and fewer complication rates [10]. However, other studies encourage more definitive treatment such as small bowel resection with the possibility of reversing the gastric bypass and opting for a gastric sleeve [8]. Due to the rarity or underreporting of this problem, no consensus has been officially reached.

Given this patient’s recurrence, she was seemingly at higher risk for failure of simple reduction. We believe that more aggressive management for recurrences of jejunal intussusception status post RYGB is appropriate, i.e., reduction with enteropexy or small bowel resection with jejunojejunostomy re-anastomosis. Enteropexy of both the antegrade and retrograde portions of the anastomotic junction should be performed [7].

Limitations

This is a single occurrence of an acute complication, which limits the generalizability of this report and our conclusion. 

## Conclusions

Intussusception is a rare or underdiagnosed complication of gastric bypass procedures that most commonly occurs 2-6 years postoperatively. Here, we present a case of recurrent intussusception 19 years after RYGB. In this patient, initial management included laparoscopic reduction of the intussusception point at the antiperistaltic junction; however, recurrence of intussusception occurred within 24 hours. While debate remains regarding the pathophysiology, and therefore treatment, of jejunal intussusception status post RYGB, given this patient’s recurrence, she was seemingly at higher risk for failure of simple reduction. More aggressive management for recurrences of jejunal intussusception status post RYGB is appropriate, i.e., reduction with enteropexy or small bowel resection with jejunojejunostomy re-anastomosis. Ideally, enteropexy of both the antegrade and retrograde portions of the anastomotic junction should be performed. In this case, the primary goal of successful resolution of the intussusception was achieved after re-anastomosis in isoperistaltic fashion without enteropexy and, despite a complicated hospital course, the anastomosis has remained patent without further intussusception at the time of discharge. 
